# Role of PAR-4 in ovarian cancer

**DOI:** 10.18632/oncotarget.4010

**Published:** 2015-05-27

**Authors:** Sonia Meynier, Marianne Kramer, Pascale Ribaux, Jean-Christophe Tille, Florence Delie, Patrick Petignat, Marie Cohen

**Affiliations:** ^1^ Department of Gynecology Obstetrics, Faculty of Medicine, University of Geneva, Switzerland; ^2^ Division of Clinical Pathology, Geneva University Hospital, University of Geneva, Switzerland; ^3^ School of Pharmaceutical Sciences, University of Geneva, University of Lausanne, Switzerland

**Keywords:** PAR-4, GRP78, ovarian cancer, apoptosis

## Abstract

Prostate apoptosis response-4 (PAR-4) is considered as a tumour suppressor due to its ability to selectively induce cell apoptosis in most cancer cells. However little is known about the role of PAR-4 in ovarian cancer. In this study, we investigated for the first time the role of PAR-4 in ovarian carcinogenesis. We showed that PAR-4 mRNA level is not significantly different between healthy and cancer ovarian cells. Immunohistochemistry on ovarian tissue showed that ovarian cancer cells are positive for PAR-4 nuclear and cytoplasmic staining whereas ovarian healthy cells are negative for PAR-4 nuclear staining. We then studied the role of PAR-4 in cell apoptosis. We determined that PAR-4 induces cell apoptosis in response to stimuli, *in vitro*, but is also involved in the relocation of GRP78 from endoplasmic reticulum to the cell surface of ovarian cancer cell line (SKOV-3 cells). *In ovo*, PAR-4 decreases ovarian tumour development and increases the response to taxol treatment. These observations suggest that PAR-4 is a very interesting therapeutic target against ovarian carcinogenesis.

## INTRODUCTION

Ovarian cancer is the leading cause of death for gynaecological cancers and the seventh highest cause of cancer death in women [1]. The International Federation of Gynaecology and Obstetrics (FIGO) classifies ovarian cancer in 4 main stages which progressively increase in severity [2]. This disease is generally detected late at stages 3 and 4 due to poor specificity of early detection methods. The standard treatment combines surgical cytoreduction with a combination of chemotherapies. Despite this aggressive approach, the 5 year survival rate is around 30% for all stages involved [3]. In order to develop new therapeutic approaches, it is important to better understand the pathological mechanisms involved in ovarian tumorigenesis.

PAR-4 is a 40kDa protein expressed in various tissues and is generally considered as a tumour suppressor [4]. This protein contains three domains: 1) two putative nuclear localization sequences, 2) a leucine-zipper domain and 3) a selective domain for apoptosis induction in cancer cells (SAC domain) [4–6]. PAR-4 is mainly localized in the cytoplasm but it can also be translocated into the nucleus in cancer cells upon cleavage by caspase-3. Once in the nucleus is inhibits NF-kB activity and activates the caspase-dependent apoptotic pathway. [7]. PAR-4 can also bind to kinases such as PKCζ or Akt and inactivate their ability to promote NFкB activation [8, 9]. It has also been shown that PAR-4 interacts with the transcription factor Wilms Tumor 1 (WT1) causing the transcriptional repression of B-cell lymphoma-2 (bcl-2) [10]. Moreover, recent findings showed that secreted PAR-4 can induce apoptosis by interacting with cell surface Glucose regulated protein 78 (GRP78) in prostate cancer cells [11]. GRP78, also known as BiP, is an endoplasmic reticulum (ER) heat shock protein in ER but it is also observed at the cell surface in many types of cancer cells [12]. Interestingly, PAR-4 itself is involved in the relocation of GRP78 from ER to the cell surface [11, 13]. The presence of GRP78 at the cell surface is extensively described in cancer cells where membranous GRP78 has functions in enhancing cell invasion, proliferation and migration in cancer cells [14, 15].

In cancer cells, the downregulation of PAR-4 activity can either be due to a decrease in mRNA levels in the cell or to the inhibition of its activity. [9, 16, 17]. In general a couple of different factors can be responsible for the inactivation of PAR-4 expression such as point mutations or promoter methylation [4]. In cancer cells, PAR-4 activity can be inhibited by 1) preventing phosphorylation, which is necessary for its proapoptotic activity [4], 2) sequestration of PAR-4 in the cytoplasm by association with Akt1 or 3) reducing caspase-3 mediated PAR-4 cleavage and preventing apoptosis [7, 18]. Therefore, different studies have sought to prevent the inhibition of PAR-4 activity. It has been shown that treatment of mice with Akt inhibitors can activate PAR-4 and reduce tumour growth [19]. Further, another study has demonstrated that the use of Bcl-2 or Src kinase inhibitors results in the activation of PAR-4 and thus of the inhibition of cell growth in pancreatic and colon cancer cells [20, 21]. Moreover, it has been shown that if PAR-4 expression is increased in cancer cells, apoptosis is induced [22] and there is a greater response to therapeutic agents and thus tumour progression is reduced [23].

These properties of PAR-4 raise questions about its role in ovarian cancer cell apoptosis and suggest PAR-4 as a potential target for new therapeutics. However, it has been demonstrated that PAR-4 could be involved in GRP78 relocation from ER to the cell surface in prostate cancer cells and trophoblastic cells [11, 13] supposing also a potential role in GRP78 relocation in ovarian cancer cells. At the moment, no previous work has been performed on the role of PAR-4 in ovarian carcinogenesis but PAR-4 has been observed in the ovary and in human ovarian cancer cell lines [7, 24]. Thus, here, we investigated for the first time the expression of PAR-4 in serous ovarian cancer cells compared to healthy ovarian cells. We explored its involvement in paclitaxel-induced apoptosis, in GRP78 relocation from ER to ovarian cancer cell surface. We finally investigated the role of PAR-4 in tumour development *in ovo*.

## RESULTS

### Presence of PAR-4 in healthy and cancer tissues

**Figure 1:**
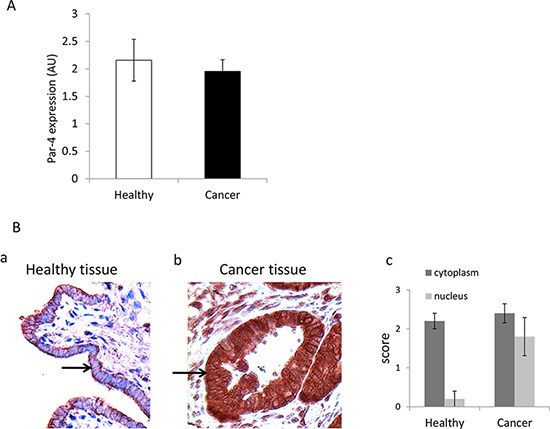
Presence of PAR-4 in healthy and cancer tissues **A.** Presence of PAR-4 mRNA in healthy and cancer tissues. qPCR analysis of PAR-4 mRNA expression in healthy and cancer cells. Two housekeeping genes were used: GAPDH and Cyclophilin A. **B.** Expression of PAR-4 in healthy and cancer tissues. Immunohistochemistry of healthy (a) and high grade serous ovarian cancer (b) tissues with control normal rabbit IgG and anti-PAR-4 antibodies (R-334). The magnification used is ×200. Arrows indicate epithelial cells. (c) Score of staining intensity established by two experts for the cytoplasm and the nucleus PAR-4 levels.

We first examined PAR-4 mRNA expression in both healthy (*n* = 12) and ovarian cancer cells (*n* = 18) by qRT-PCR. As shown in Figure 1A, PAR-4 mRNA expression doesn't significantly differ between cells purified from healthy and cancer tissues. We then evaluated the PAR-4 location in healthy and cancer tissues (Figure 1Ba and 1Bb). The intensity of staining of cytoplasm and nucleus is scored as absent (0), weak (1), moderate (2) and intense (3) (Figure 1Bc). As shown in Figure 1B, PAR-4 is mainly immunolocalised in the cytoplasm in healthy tissues whereas it is found in both the nucleus and the cytoplasm in high grade serous ovarian cancer tissues.

### Effect of PAR-4 on apoptosis

**Figure 2:**
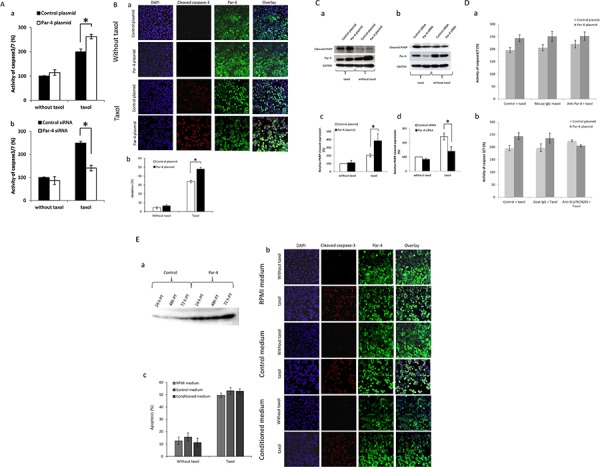
Effect of PAR-4 levels on cell apoptosis **A.** Analysis of caspase 3/7 activity. SKOV-3 cells are transfected with control or PAR-4 expressing plasmid (a) and with control or PAR-4 siRNA (b) and treated or not with 100 nM of taxol. After 24 hours, luminescent assay is performed to measure caspase3/7 activity. Quantitative results are presented in Figures a and b. **B.** Apoptosis scoring of active caspase-3. SKOV-3 cells are transfected with control or PAR-4 expressing plasmid. 24 hours after, SKOV-3 cells are seeded and treated or not with 100 nM of taxol for 24 hours. SKOV-3 cells are washed, fixed and then, incubated with anti-PAR-4 antibodies (R-334) and active caspase-3 antibodies. Then, cells are incubated for 1 hour with Alexa Fluor-488 donkey anti-rabbit IgG or Alexa Fluor-568 donkey anti-mouse IgG. The nucleus is stained with DAPI. SKOV-3 cells are analysed with LSM 510 META. The magnification used is ×200 (a). The percentage of apoptotic cells is calculated as follows: Apoptosis % = number of active caspase 3 positive cells/number of nuclei (b). **C.** Analysis of cleaved PARP by Western Blot. SKOV-3 cells are transfected with control or PAR-4 expressing plasmid (a) and with control or PAR-4 siRNA (b) and treated or not with 100 nM of taxol. After 48 hours, cell extracts are collected. Western Blot is performed and probed with anti-cleaved-PARP and anti-GAPDH antibodies. Bands of western blot are revealed by an ECL method, scanned and quantified by the Kodak 1D image analysis software. The cleaved-PARP band intensity is normalized to GAPDH and is expressed in percentage in function of control plasmid without taxol (c, d). **D.** Analysis of caspase3/7 activity with inhibition of PAR-4. SKOV-3 cells transfected with control or PAR-4 expressing plasmid are treated or not with 100 nM of taxol and 2.5 ug/ml of anti-PAR-4 antibodies (a) or anti-GRP78 antibodies (N20) (b) or IgG control for 24 hours. After 24 hours, caspase3/7 activity is examined by luminescent assay 24 post-treatment with antibodies. Quantitative results are presented in Figures a and b. **E.** Active caspase-3 staining in SKOV-3 cells treated or not with conditioned media. SKOV-3 cells are transfected with control or PAR-4 expressing plasmids. The culture supernatant is collected at 24, 48 h and 72 h post transfection (PT), and centrifuged at 14 000 rpm for 10 min. The supernatant is then aliquoted and frozen until use. Western blot is performed to evaluate the presence of secreted PAR-4 in medium (a). SKOV-3 cells are transfected with control or PAR-4 and GRP78 expressing plasmids. The efficiency of transfection is confirmed by western blot (data not shown). 24 hours after transfection, SKOV-3 cells are seeded in Lab-tek chamber. 24 hours after, SKOV-3 cells are treated or not with 100 nM of taxol and with 72 h PT conditioned medium for 24 hours. SKOV-3 cells are washed, fixed and then, incubated with anti-PAR-4 antibodies (R-334) and active caspase-3 antibodies. Then, cells are incubated for 1 hour with Alexa Fluor-488 donkey anti-rabbit IgG or Alexa Fluor-568 donkey anti-mouse IgG. The nucleus is stained with DAPI. SKOV-3 cells are analysed with LSM 510 META. The magnification used is ×200 (b). The percentage of apoptotic cells is calculated as follows: Apoptosis % = number of active caspase 3 positive cells/number of nuclei (c).

Based on previous studies showing that PAR-4 is involved in apoptosis [4], we decided to investigate the effect of PAR-4 on apoptosis of an ovarian cancer cell line SKOV-3 under normal conditions or after paclitaxel treatment. Under basal condition, PAR-4 levels do not influence the activity of caspase-3/7 (Figure 2A), the active caspase-3 level staining (Figure 2B) or the level of cleaved PARP (Figure 2C). However, under taxol treatment, PAR-4 overexpression increases the activity of caspase3/7 (Figure 2A), active caspase-3 staining (Figure 2B) and the cleaved-PARP expression (Figure 2C). Similarly when PAR-4 expression is decreased, the activity of caspase3/7 (Figure 2A) and the cleaved-PARP expression (Figure 2B) were decreased. These results are confirmed in another ovarian cancer cell line, A2780 cell line (Supplementary Figure S1).

In addition, we observe that PAR-4 is mainly localised in cytoplasm in non-treated cells and is found in cytoplasm and nuclei after taxol treatment (Figure 2B).

Based on a study that showed the secreted form of PAR-4 could bind membranous GRP78 and induce apoptotic pathway [11], we assessed the effect of secreted PAR-4 on cell apoptosis in presence of anti-PAR-4 and anti-GRP78 antibodies (N20) (Figure 2D). Anti-N20 antibody is used to compete with secreted PAR-4 for the binding to membranous GRP78 [11]. Under taxol treatment, the activity of caspase-3/7 does not significantly change between cells treated with anti-PAR-4 antibodies or control mouse IgG in both the cells transfected with the control plasmid and the PAR-4 expression plasmid (Figure 2D). The treatment of SKOV-3 cells with anti-GRP78 antibodies (N20) does not influence the activity of caspase-3/7 for SKOV-3 cells transfected with control plasmid or PAR-4 plasmid compared to untreated cells or IgG treated cells respectively (Figure 2D).

To complete this finding, we tested the effect of secreted PAR-4 using PAR-4 conditioned media on SKOV-3 cell apoptosis. In order to do this, SKOV-3 cells were transfected with PAR-4 plasmid or control to prepare conditioned medium. SKOV-3 cells in which PAR-4 and GRP78 expression were increased were treated with control or PAR-4 conditioned medium. Then, ICC for active caspase-3 was performed to detect apoptosis. PAR-4 conditioned medium has no effect on SKOV-3 cells apoptosis compared to the control medium either in presence of taxol or not (Figure 2E).

These results suggest that secreted PAR-4 is not involved in SKOV-3 cells apoptosis.

### Colocalization of PAR-4 and GRP78

**Figure 3:**
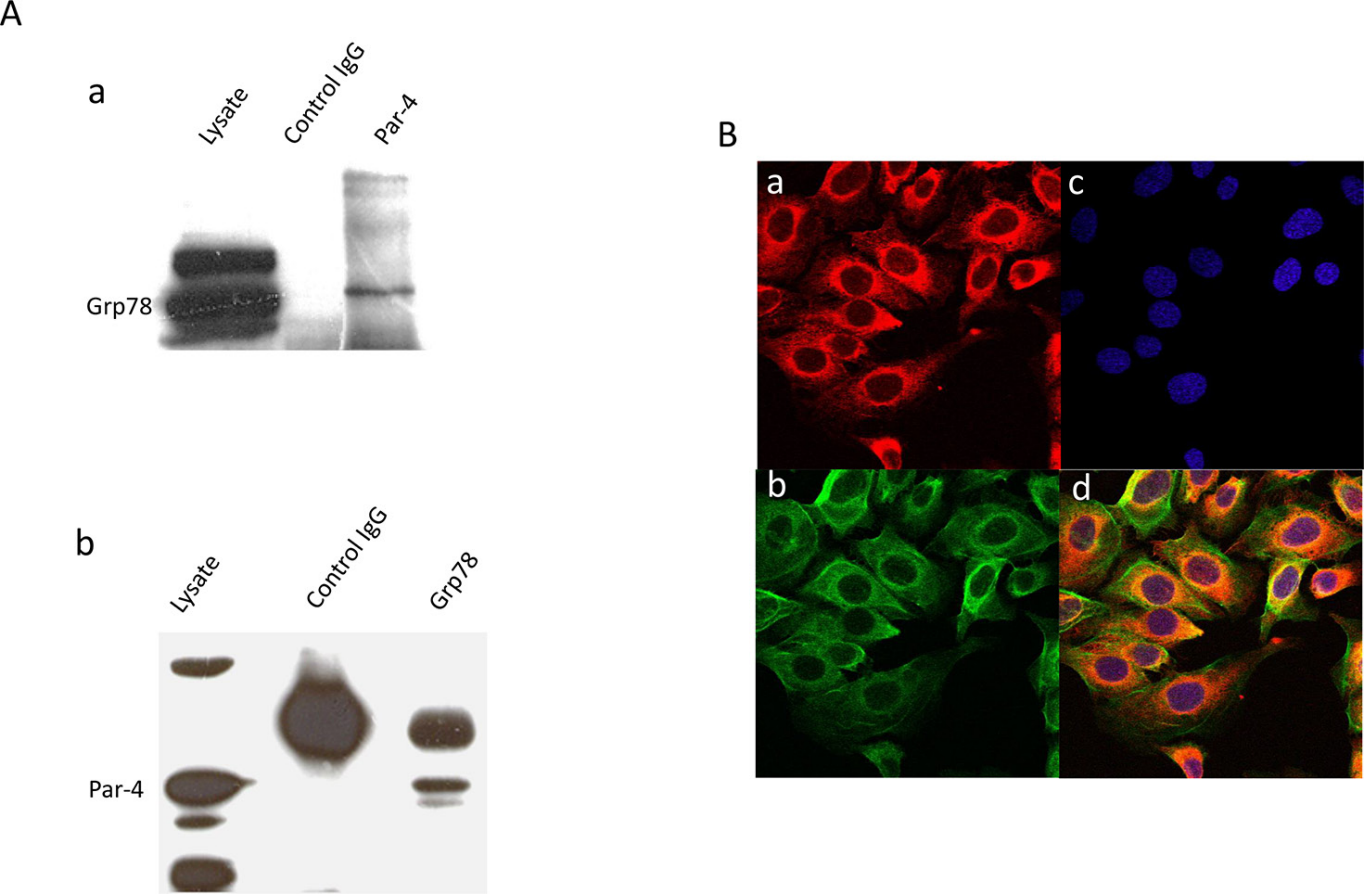
Colocation of PAR-4 and GRP78 **A.** Co-immunoprecipitation. a. Western blot analysis of SKOV-3 cell lysate immunoprecipitated with anti-PAR-4 antibodies and blotted with GRP78 (Gl-19) antibodies. b. Western blot analysis of SKOV-3 cell lysate immunoprecipitated with anti-GRP78 (Gl-19) antibodies and blotted with anti-PAR-4 antibodies. **B.** Immunofluorescence. Immunofluorescence assay of SKOV-3 cells is performed with anti-GRP78 (N20) (a), and anti-PAR-4 antibodies (b). The nucleus is stained with Dapi (c). The cells are analysed with LSM 510 META. The magnification used is ×400. In the merged images (d), the colocation is shown in yellow.

Previously, it has been shown that PAR-4 could form a complex with GRP78 in the ER of prostate cancer cells [11]. Here, we decided to confirm the possibility that PAR-4 could bind to GRP78 in SKOV-3 cells by co-immunoprecipitation and western blot analysis (Figure 3A). We then investigated the colocalization of PAR-4 and GRP78 in SKOV-3 cells by immunofluorescence microscopy (Figure 3B). PAR-4 and GRP78 are mainly present in the cytoplasm. The colocalized signal in yellow (Figure 3Bd) is found in the cytoplasm and mainly in perinuclear region.

### Effect of PAR-4 on GRP78 relocalization at the ovarian cancer cell surface

**Figure 4:**
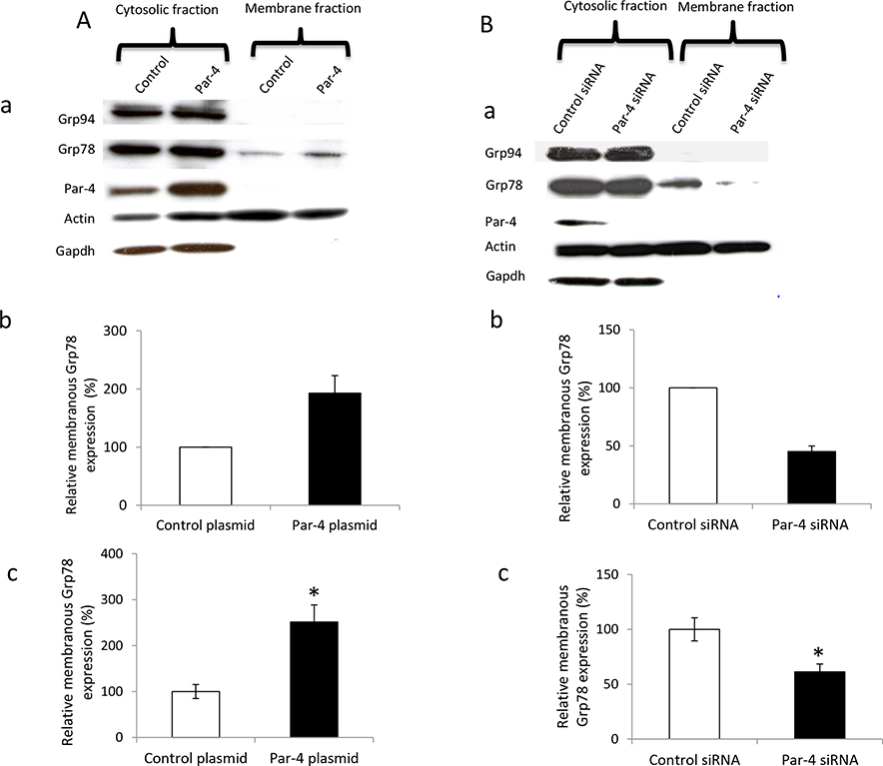
Effect of PAR-4 expression on GRP78 relocation in ovarian cancer cell surface **A.** Effect of PAR-4 overexpression on GRP78 relocation. a. SKOV-3 cells are transfected with control or PAR-4 expressing plasmid. Western blot is done and probed with anti-GRP78, anti-PAR-4, anti-Actin, as loading control, and anti-GAPDH, as cytosol control, antibodies. b. Bands of western blot are revealed by an ECL method, scanned and quantified by the Kodak 1D image analysis software. The GRP78 band intensity is normalized to Actin and is expressed in percentage in function of control plasmid. c. Transfected SKOV-3 cells are seeded at 20 000 cells/well in 96-well plate. After 48 hours, cells are incubated with anti-GRP78 antibodies and then with HRP conjugated goat anti-rabbit IgG antibody. The results are expressed as the relative ration of membranous GRP78 over total GRP78 (*p < 0.05, Student *t*-test). **B.** Effect of PAR-4 downregulation on GRP78 relocation. a. SKOV-3 cells are transfected with control or PAR-4 siRNA. Western blot is done and probed with anti GRP78, anti-PAR-4, Actin and GAPDH antibodies. b. Bands of western blot are revealed by an ECL method, scanned and quantified by the Kodak 1D image analysis software. The GRP78 band intensity is normalized to Actin and is expressed in percentage in function of control plasmid. c. Transfected SKOV-3 cells are seeded at 20 000 cells/well in 96-well plate. After 48 hours, cells are incubated with anti-GRP78 antibodies and then with HRP conjugated goat anti-rabbit IgG antibody. The results are expressed as the relative ration of membranous GRP78 over total GRP78 (*p < 0.05, Student *t*-test).

We first determined that PAR-4 has no impact on GRP78 expression or secretion (Supplementary Figure S2). Then, we evaluated whether an increase or decrease in PAR-4 levels could affect the location of GRP78 in SKOV-3 cells. Figure 4A shows that overexpression of PAR-4 increases the membranous level of GRP78 in SKOV-3 compared to control plasmid transfected cells. Similarly, a decrease in PAR-4 expression leads to a decrease in cell surface expression of GRP78 (Figure 4B). Glucose regulated protein 94 (Grp94), an ER molecular chaperone, is not present at the cell surface in both conditions suggesting that PAR-4 is involved in GRP78 relocation but not in the relocation of all ER proteins.

### Role of PAR-4 on tumour development

**Figure 5:**
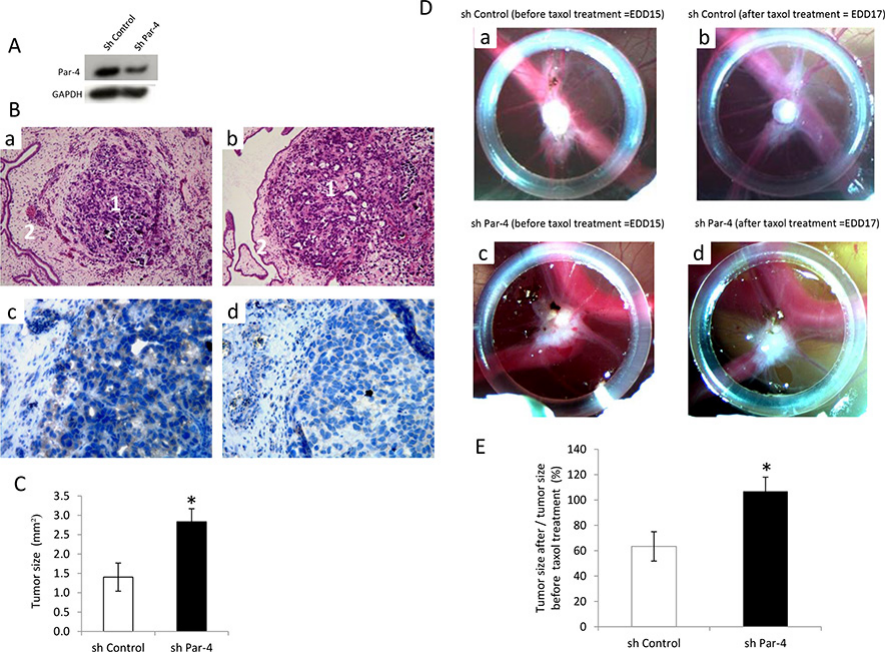
Influence of PAR-4 on the efficiency of taxol on tumour development **A.** Western blot analysis of PAR-4 levels after transfection of SKOV-3 cells with shControl or shPAR-4. **B.** HE staining of shControl tumour (a) and shPAR-4 tumour (b) tissues. The magnification used is ×200. Numbers 1 indicate tumours and numbers 2 show the chicken membrane. PAR-4 staining of shControl tumour (c) and shPAR-4 (d) tissues. The brown colour represents the PAR-4 expression. The magnification used is ×400. **C.** Evaluation of the tumour size before taxol treatment on EDD 15 (*n* = 10 for shControl and *n* = 14 for shPAR-4) using ImageJ software. **D.** On EDD8, shControl SKOV-3 cells (a) or shPAR-4 SKOV-3 cells (c) suspension are placed into the silicon O-ring for 4 days to allow tumour growth. On EDD15, shControl (a) or shPAR-4 tumours (c) are treated topically with 25 μl of 25 μM of taxol for 2 days. On EDD17, tumour size of shControl (b) and shPAR-4 (d) is monitored using a Wild Heerbrugg M3Z microscope at 10× magnification with a Lumenera INFINITY2–1 CDD camera with Infinity Capture Software. **E.** Evaluation of the tumour size after taxol treatment (*n* = 10 for shControl and *n* = 14 for shPAR-4) using ImageJ software. The size of tumours on EDD 17 is measured and the average is done.

We then examined the role of PAR-4 on tumour development using a CAM model (Figure 5). We have established a stable SKOV-3 cell line where Par-4 is diminished to investigate the effect of endogenous PAR-4 on tumour development and the response of the tumour to taxol treatment.

First, PAR-4 silencing efficiency in SKOV-3 cells is evaluated by Western Blot (Figure 5A). Then, tumours are observed by a haematoxylin and eosin (HE) staining and the decrease in PAR-4 level in the shPAR-4 tumours compared to shControl tumours are checked by immunohistochemistry (Figure 5B).

We first compared tumour size of shControl and shPAR-4 at embryonic development day (EDD) 15. As shown in Figure 5C, tumours with decreased PAR-4 level are significantly larger than control tumours.

We then examined the effect of taxol on shControl tumours or shPAR-4 tumours (Figure 5B). In presence of 25 μM of taxol, the size of shControl tumours decrease about 40% while PAR-4 tumours doesn't change in size despite taxol treatment (Figure 5E).

## DISCUSSION

PAR-4 is a tumour suppressor with intracellular functions in both the cytoplasm and the nucleus [4]. In cytoplasm, PAR-4 can bind protein kinases and inhibit cell survival pathways [4]. Furthermore, PAR-4 can be translocated to the nucleus where it inhibits the NF-ĸB-mediated transcription of survival genes or the action of TOPO1 [4]. However, PAR-4 mediated-apoptosis could be impeded in cancer cells either by a downregulation of PAR-4 expression and/or by decreased activity of PAR-4 due to its non-phosphorylation or its sequestration in cytoplasm preventing its entry into the nucleus [4]. Here, we examined, for the first time, the levels of PAR-4 mRNA and its location in both healthy and high grade serous cancerous human ovarian tissues. The levels of PAR-4 mRNA and protein expression do not significantly differ between healthy and high grade serous ovarian cancer cells. It is mainly localized in the cytosol in healthy ovarian tissues while its expression is seen in both cytoplasm and nucleus of ovarian cancer cells suggesting that PAR-4 could have a pro-apoptotic activity in ovarian cancer cells as shown in different cancer cells.

Thus, we decided to explore the role of PAR-4 in apoptosis of ovarian cancer cell lines. In contrast to most cancer cell lines in which ectopic PAR-4 translocates to the nucleus and induces apoptosis, in ovarian cancer cell lines ectopic PAR-4 is mainly localized to the cytoplasm and does not induce apoptosis. However, upon taxol treatment, PAR-4 translocates to the nucleus and increases taxol response in SKOV-3 cells suggesting that taxol-induced apoptosis may be a result of the movement of PAR-4 into the nucleus. The “apoptosis-sensitizing” function of PAR-4 has already been described in hormone-responsive cancer cells [5]. It is generally attributed to its sequestration in the cytoplasm. However, treatment with apoptosis inducer translocates PAR-4 into the nucleus and induces apoptosis. In taxol-treated SKOV-3 cells, nuclear localization of PAR-4 is associated with increased caspase 3 activity. This is in accordance with the hypothesis that caspase 3 is responsible for PAR-4 cleavage and thus its entry into nucleus to induce apoptosis [7].

These observations made in ovarian cancer cell lines suggest that nuclear location of PAR-4 in tissue could be due to a stress activating a pathway leading to entry of PAR-4 into the nucleus *in vivo*. It is also possible that these ovarian cancer cell lines are not fully representative of serous ovarian cancer.

We then investigated the role of secreted PAR-4 in apoptosis of SKOV-3 cells. In some cancer cells, it was shown that interaction of extracellular PAR-4 with cell-surface GRP78 induces apoptosis in a FADD-dependent manner [11]. However, in SKOV-3 cells, and despite the fact that ectopic PAR-4 increases GRP78 relocation at the cell surface, treatment of SKOV-3 cells with PAR-4 conditioned medium does not induce apoptosis, even in taxol treated cells. Moreover, the use of GRP78 or PAR-4 antibodies in culture medium to interfere within the interaction between secreted PAR-4 and membranous GRP78 does not impact PAR-4 induced apoptosis in taxol-treated cells suggesting that secreted PAR-4 does not play a significant role in “apoptosis-sensitizing” function of ovarian cancer cells.

We also investigated the role of endogenous PAR-4 in the relocation of GRP78 from the ER to the cell surface as it was described in other cancer cells [11, 13]. In physiological conditions, GRP78 is present in ER and facilitates the folding and the assembly of newly synthesized proteins, prevents protein aggregation during stress conditions and participates in the regulation of calcium homeostasis [12]. Although GRP78 has been described as an ER luminal protein, it can also be found in other cellular locations. In stressed cells or in most cancer cells (including ovarian cancer cells [12]), GRP78 is overexpressed and found at the cell surface. At the cell surface, it can bind other proteins such as alpha-2-macroglobulin or cripto and thus induces signal pathways involved in tumorigenesis [25, 26]. Membranous GRP78 has also the potential to form a complex with PAR-4 or Kringle 5 resulting in the induction of the apoptosis pathway [11, 27]. However, in this study, we demonstrate that membrane GRP78 does not seem to play a major role in PAR-4-induced apoptosis in ovarian cancer cells. Considering the significance of cell surface GRP78, it was important to investigate the role of endogenous PAR-4 in GRP78 relocation at cell surface of ovarian cancer cells.

Thus, we first determined that PAR-4 and GRP78 colocalize in the ovarian cancer cell line SKOV-3 by immunofluorescence and further substantiated a possible interaction between PAR-4 and GRP78 by immunoprecipitation. It is found that neither increasing nor decreasing PAR-4 expression has an impact on total GRP78 levels indicating that GRP78 expression is independent of PAR-4. Thus, we focused on the role of PAR-4 in GRP78 relocation, and find that PAR-4 is involved in the relocation of GRP78 from ER to the cell surface in SKOV-3 cells, as indicated by the decreased amount of membranous GRP78 with the decreased PAR-4 expression. To supplement this finding, we observed a significant correlation between levels of PAR-4 mRNA and membranous GRP78 expression in purified ovarian cells strengthening the hypothesis that PAR-4 participates in the GRP78 transport in ovarian cancer cells (Supplementary Figure S3). It has also been shown that GRP78 could be secreted into human peripheral circulation and activates pro-survival pathways [28, 29]. We analysed the secreted form of GRP78 in relation to the amount of PAR-4 expressed in the cells and saw that changes in PAR-4 expression don't influence the level of secreted GRP78 suggesting that the role of PAR-4 in GRP78 relocation is limited to its transport to the cell membrane.

PAR-4 could be a potential therapeutic target in ovarian cancer due to its important role in apoptosis *in vitro*. On the other hand, it could also induce relocation of GRP78 to cell surface where this last protein could mediate some signalling pathways involved in tumorigenesis [29]. To support PAR-4 as a potential therapeutic target in ovarian cancer, we decided to investigate the role of endogenous PAR-4 on ovarian tumour development using a shPAR-4 SKOV-3 cell line and the *in ovo* CAM-assay. We found that the downregulation of PAR-4 increased ovarian tumour growth. It also reduces the effect of taxol treatment on tumours. These results support the essential role of PAR-4 in apoptosis of ovarian cancer cells.

In summary, our study suggests that PAR-4 triggers apoptosis under apoptosis stimuli but also participates in the relocation of GRP78 to the cell surface in ovarian cancer cells. Using an *in ovo* model, we demonstrate that reducing PAR-4 increases ovarian tumour development and decreases the response to taxol suggesting that PAR-4 overexpression could be an interesting pathway to target apoptosis and to decrease tumour development in ovarian cancer.

## MATERIALS AND METHODS

### Reagents

Monoclonal mouse anti-human PAR-4 (3G9H7), polyclonal rabbit anti-human PAR-4 (R334), polyclonal mouse anti-human GAPDH (V18), monoclonal mouse anti-human Calnexin (AF18), monoclonal mouse anti-human cleaved PARP-1, normal mouse IgG, normal rabbit IgG and polyclonal goat anti-human GRP78 (C-20 and N-20), donkey anti-goat IgG-horseradish peroxidase (HRP) and goat anti-mouse IgG-HRP and control and PAR-4 shRNA plasmid as well as control and PAR-4 siRNA were purchased from Santa Cruz Biotechnology (San Diego, CA, USA). Polyclonal rabbit anti-human GRP78 (Gl19) was purchased from Sigma Aldrich (St Louis, MO, USA). Mouse active caspase-3 antibody was purchased from R&D Systems (Minneapolis, USA). Monoclonal mouse anti-human actin (clone C4) come from Millipore (Zug, Switzerland). Goat anti-rabbit IgG-HRP was purchased from BioRad (BioRad, Marnes-la-Coquette, France). Alexa Fluor-568 donkey anti-goat IgG, Alexa Fluor-488 donkey anti-rabbit IgG and or Alexa Fluor-568 donkey anti-mouse IgG were purchased from Biolegend (San Diego, CA, USA).

The PAR-4 expressing plasmid was kindly provided by Professor Rangnekar Vivek (University of Kentucky, USA).

### Ethics statement

This research has been approved by the departmental ethics committee of maternity and paediatrics, University Hospital of Geneva. Informed written consent was obtained from all patients before their inclusion in the study (CER09–052).

### Purification of ascites cells

Ascites (*n* = 18) were centrifuged at 600 g for 8 minutes. The pellet was resuspended in Hanks Balanced Salt Solution (HBSS, Gibco, Invitrogen, Basel, Switzerland) containing 25 mM Hepes (Gibco, Invitrogen, Basel, Switzerland) and 0.05 mg/ml gentamicin (Invitrogen, Basel, Switzerland) and centrifuged at 600 g for 8 minutes. The resulting pellet was resuspended in HBSS-Hepes 20 mM and filtered through a 100 μm mesh (BD Biosciences, San Jose, USA). The filtrate was resuspended with Dulbecco's Modified Eagles Medium (DMEM, Sigma Aldrich, St Louis, MO, USA) containing 10% fetal bovine serum (FBS, Biochrom AG, Oxoid AG, Basel, Switzerland) and 25 μg/ml plasmocin (InvivoGen, San Diego, CA, USA). Blood cells were eliminated by performing a Percoll (GE Healthcare, Zurich, Switzerland) gradient and centrifuged at 1200 g for 20 minutes (10%, 30%, 40%, and 70% of Percoll diluted in HBSS). Then, the cellular ring between layers 40% and 20% of Percoll was collected, diluted in DMEM and centrifuged at 600 g for 8 minutes. The pellet was resuspended and cells were counted and seeded in 3 cm dish.

### Purification of cancer and healthy cells

Ovarian tissue (*n* = 15) was digested with 4 mg/ml dispase (Gibco, Invitrogen, Basel, Switzerland) in HBSS-Hepes (filtered on 22 μm) containing 1 μg/ml Dnase (Roche, Diagnostics GmbH, USA) for 30 minutes at 37°C. Ovarian tissue and supernatant were put in 10 cm dish and tissue was scrubbed with a scalpel. Then, the supernatant was collected, neutralized with 5% FBS, filtered through a 100 μm mesh (BD Biosciences, San Jose, USA) and centrifuged at 800 g for 8 minutes. The resulting pellet was resuspended in DMEM-10%FBS-0.05 mg/ml gentamicin and cells were seeded in 3 cm dish. Cells were characterized by PCR (CD90, HE4, PAX8, cytokeratin 8, cytokeratin 19, cytovillin) and by immunocytochemistry (cytokeratin 7, cytokeratin 18, cytokeratin 19, vimentin, p53). Cells were used before passage 5.

### qRT-PCR

Expression of PAR-4 mRNA has been investigated on purified cells from borderline tumor (*n* = 3), ovarian healthy (*n* = 12) and high grade serous ovarian cancer (*n* = 18) tissues or ascites. Reverse transcription was performed with 1 μg of total RNA in a final volume of 20 μl using High Capacity cDNA Reverse Transcription Kit (Applied biosystems, Foster city, USA). The quantitative detection of the PCR product was performed using KAPA SYBR FAST Universal qPCR kit (KAPA biosystems, Boston, USA), with the iCycler iQ System (Bio-Rad). The relative expression of PAR-4 mRNA was normalized to the housekeeping genes GAPDH and Cyclophilin A. The experiment was carried out in triplicate.

Oligonucleotide primers for qPCR are listed in Table 1.

**Table 1:** Oligonucleotide list

	foward (5′-3′)	reverse (5′-3′)
Par-4	CTGCCGCAGAGTGCTTAGAT	CATCTTCTCGTTTCCGCTCT
GAPDH	CGACCACTTTGTCAAGCTCA	CCCTGTTGCTGTAGCCAAAT
Cyclophilin A	TACGGGTCCTGGCATCTTGT	CCATTTGTGTTGGGTCCAGC

### Immunohistochemistry

Ovarian cancer tissues were rapidly washed with 0.1 M phosphate buffered saline (PBS) at pH 7.4 and fixed for 4–12 hours in 4% buffered formalin at 4°C. The specimens were then dehydrated in ethanol and embedded in paraffin wax.

5 sections of human healthy ovarian tissue and 5 sections of human ovarian cancer tissue were deparaffinised and rehydrated through graded ethanol. Antigen retrieval was performed by microwave pre-treatment in 10 mmol/l citrate buffer (pH 6.0) for 5 minutes four times, followed by cooling in a cold water bath. Non-specific binding was blocked with 3% (v/v) bovine serum albumin (BSA) in PBS for 30 minutes at room temperature. The sections were incubated with polyclonal rabbit anti-human PAR-4 (R-334) or with control IgG (dilution 1/500 in 3% BSA-PBS) overnight at 4°C. Sections were then washed with PBS and incubated with goat anti-rabbit IgG-HRP (dilution 1/500) for 1 hour. After washing, sections were stained with diaminobenzidine (DAB) chromogen system (Dako, Baar, Switzerland). The stained tissue was scored independently by 2 experts. The intensity of cytoplasm and nucleus staining was scored as absent (0), weak (1), moderate (2) and intense (3).

### Cell culture

SKOV-3 human ovarian cancer cells (ATCC HTB-77™) A2780 human ovarian cancer cells (ECACC 93112519) were cultured at 37°C under 5% CO_2_ in Roswell Park Memorial Institute medium 1640 (RPMI 1640, Gibco, Invitrogen, Basel, Switzerland) containing 10% fetal bovine serum and 0.05 mg/ml gentamicin (Gibco, Invitrogen, Basel, Switzerland).

### Transfection

For siRNA transfection, 1.2 × 10^6^ SKOV-3 or A2780 cells were plated in a 10 cm dish and immediately transfected with 10 nM of control or PAR-4 siRNA. 16 hours after transfection, cells were trypsinized and seeded for 48 hours for cell ELISA, cell viability, invasion assays as well as apoptosis analysis. Transfection was performed using INTERFERin (Polyplus, Illkrich, France) following the reverse transfection protocol provided by the manufacturer. For plasmid transfection, 1.2 × 10^6^ cells were plated and incubated at 37°C for 16 hours. Cells were transfected with control or PAR-4 expressing plasmid or GRP78 expressing plasmid using jetPEI (Polyplus, Illkrich, France) following the manufacturer instructions. 16 hours after transfection, cells were trypsinized, seeded in appropriate dish and incubated for 48 hours. The experiment was carried out in triplicate.

### Establishment of stable cell lines

8 × 10^5^ SKOV-3 cells were seeded in 6 cm dish. The next day, the SKOV-3 cells were transfected with control or PAR-4 shRNA plasmid using jetPEI reagent following the manufacturer's instructions. At 48 h post-transfection, SKOV-3 cells, which have integrated plasmid, were selected with RPMI-1640 complemented with 4 ug/ml of puromycin (Sigma Aldrich, St Louis, MO, USA). Every 2 days, the medium was changed to select transfected cells.

### Activity of caspase-3/7

Transfected cells were seeded at 3 × 10^4^ cells/well in a 96-well plate for 24 hours. Cells were then treated or not with 100 nM of taxol and incubated with 2.5 ug/ml of anti-PAR-4 antibodies, anti-GRP78 antibodies (N20) or control IgG for 24 hours. The activity of caspase-3/7 was measured with a Caspase-Glo^R^ 3/7 Assay kit (Promega, Madison, USA), according to the manufacturer's instructions, using GloMaxTM 96 Microplate Luminometer (Promega, Madison, USA). The experiment was carried out in triplicate.

### Immunofluorescence and apoptosis scoring

SKOV-3 cells were seeded into Lab-tek chamber slide 8 wells (2 × 10^4^ cells/well) and cultured as described above. After 24 hours, cells were washed in Phosphate Buffer Saline (PBS), fixed in 4% Paraformaldehyde (PFA) for 15 minutes and washed 3 times in PBS. Cells were permeabilized with ice cold methanol for 3 minutes and washed again three times in PBS. Afterwards, non-specific sites were blocked with 3% BSA in PBS for 30 minutes and incubated overnight at 4°C with polyclonal rabbit antibodies against PAR-4 (R-334, dilution 1:100) or polyclonal goat antibodies against GRP78 (C-20, dilution 1:100) or human/mouse active caspase-3 antibodies (dilution 1:800). The day after, cells were washed three times in PBS and blocked again with PBS-3%BSA for 30 minutes. Cells were incubated for 1 hour with Alexa Fluor-488 donkey anti-rabbit IgG or Alexa Fluor-568 donkey anti-goat IgG or Alexa Fluor-568 donkey anti-mouse IgG. Cells were washed 5 times in PBS before the addition of mounting medium containing Dapi-Fluoromount-G™ (SouthernBiotech, Birmigham, USA). A glass coverslip was put on slide and the sample was analysed with LSM 510 META (Carl Zeiss Microscopy, LLC, USA).

To evaluate the number of apoptotic cells, an activated caspase-3 labeling apoptotic index was performed. For each condition, 2 fields with a magnification of 200 were counted for DAPI staining and active caspase-3 staining. Then, the percentage of apoptosis is calculated as follows: number of cells with positive active caspase-3 staining over total number of cells (DAPI staining) X100.

### Co-immunoprecipitation

2 × 10^6^ SKOV-3 cells were plated in 10 cm-dish for 24 hours before being lysed in a buffer containing 10 mM Tris-HCl, 1% Triton, 150 mM NaCl, 1 mM Na_3_VO_4_, 0.5% NP-40, and protease inhibitors cocktail (Roche, Basel, Switzerland). Protein concentration was determined by BioRad assay (BioRad, Marnes-la-Coquette, France). 400 μg of total proteins were subjected to preclearing with 1 μg of IgG antibodies and 10 μL of red protein G sepharose beads (Sigma Aldrich, St Louis, MO, USA) for 1 hour. The sample was centrifuged at 8000 g for 5 minutes. Then, the lysate was immunoprecipitated overnight at 4°C with 4 μg of antibodies against target protein or IgG control antibodies. Then, 50 μL of red protein G sepharose beads were added for 1 hour. The immunoprecipitate was washed with lysis buffer and the entire volume was subject to Western blotting analysis.

### Western blot

Proteins (8 μg for subcellular fraction or 20 μg for total proteins) were fractionated by 10% SDS-PAGE and transferred to nitrocellulose membrane for immunoblot analysis using mouse monoclonal anti-PAR-4 antibodies (3G9H7, dilution 1:3000), rabbit polyclonal anti-GRP78 antibodies ( Gl19, dilution 1:3000), mouse monoclonal anti-actin antibodies (dilution 1:5000) or mouse polyclonal anti-GAPDH antibodies (dilution 1:5000). Secondary antibodies were anti-mouse-HRP (dilution 1:3000) and anti-rabbit-HRP (dilution 1:3000). All primary antibodies were diluted in 5% PBS-milk and incubated overnight at 4°C for and secondary antibodies were incubated for 1 hour at room temperature. Specific signal was detected by chemiluminescence using the ECL kit (GE Healthcare, Zurich, Switzerland). The experiment was carried out in triplicate.

### Subcellular fractionation

1 × 10^6^ SKOV-3 cells were plated into 10 cm dish and transfected as described above. After 48 hours, cells were harvested in 0.5 ml lysis buffer and cell lysate was passed through a 25 G needle 10 times and centrifuged at 720 g for 5 minutes to obtain the pelleted nuclear fraction. The supernatant was collected and centrifuged at 10 000 g for 10 minutes to get the pelleted mitochondrial fraction. At this step, the supernatant contained both cytosolic and membrane fractions. To separate these two fractions, the supernatant was centrifuged at 40 000 g for 1 hour. The resulting pellet was the membrane fraction and supernatant was the cytosolic one.

All fractions, except the cytosolic one, were washed with subcellular fraction buffer (250 mM Sucrose, 20 mM HEPES (pH 7.4), 10 mM KCl, 1.5 mM MgCl_2_, 1 mM EDTA, 1 mM EGTA, 1 mM DTT and protease inhibitors cocktail (Roche, Basel, Switzerland)) and passed through a 25 G needle 10 times. The different fractions were centrifuged again and resuspended in lysis buffer supplemented with 10% glycerol and 0.1% SDS. The cytosolic fraction was concentrated on a centricon (Millipore, Zug, Switzerland). The protein concentration in each fraction was determined by BioRad assay. The experiment was carried out in three times.

### Cell ELISA

Transfected SKOV-3 cells or purified ovarian cells were seeded at 2 × 10^4^ and 3 × 10^4^ cells/well respectively in a 96-well plate. Cells were either incubated directly with the primary antibodies or washed, fixed (3% PFA in PBS), washed, permeabilized (0.2% triton in PBS) and pre-incubated with culture medium (30 minutes) before incubation with anti-GRP78 antibodies (Sigma Aldrich, St Louis, MO, USA) for 45 minutes (dilution : 1/200). To remove the unbound antibodies, cells were washed four times in culture medium, and then incubated 30 minutes at 4°C with HRP conjugated goat anti-rabbit IgG antibody (dilution : 1/500). After incubation, cells were washed as described above and the substrate 3, 3′, 5, 5′-tertramethyl benzidine (MTT) (R&D systems, Minneapolis, USA) was added. The reaction was stopped by adding 0.2 M sulphuric acid. Absorbance was read at 450 nm on a microplate reader. These experiments were carried out in triplicate, three times.

### Tumor development and treatment on chick chorioallantoid membrane (CAM) Chick embryo culture

On the first embryonic development day (EDD1), fertilized eggs (animal facility of the University of Geneva, Geneva, Switzerland) were incubated at 38°C with 80% relative humidity and periodic rotation.

On EDD4, rotation was stopped and eggs were drilled at their narrow apex. The hole was closed with adhesive tape. Incubation was carried out until use.

On EDD8, the hole at the apex was enlarged and after gently scratching of the membrane with a needle tip, a silicon O-ring (Apple Rubber products inc., Lancaster, USA) was placed onto a blood vessel crossing. SKOV-3 cells were resuspended at 2 × 10^6^ cells in 35ul of a mixture containing 50% of RPMI-1640 (containing 10% fetal bovine serum and 0.05 mg/ml gentamicin) and 50% of BD Matrigel™ as a basement membrane matrix (BD biosciences, Bedford, USA). Then, this suspension was placed into the silicon O-ring. The hole in the egg shell was hermetically covered with Parafilm^®^ and eggs were returned to the incubator.

On EDD 15, tumor growth was monitored using a Lumenera INFINITY2–1 CDD camera with Infinity Capture Software. Then, tumor was treated topicallywith 25 μl of 25 μM taxol diluted in complete medium for 2 days into the incubator.

On EDD 17, tumor growth was again monitored. The size of tumor was analyzed with the ImageJ software.

### Statistical analysis

Data were expressed as mean ± SEM for n different samples. *P* values were calculated using the Student's *t* test and the *p* value < 0.05 was considered significant. A Pearson's correlation coefficient was used to evaluate the correlation between PAR-4 mRNA and relative expression of membrane GRP78.

## SUPPLEMENTARY DATA


