# Supplementary material for: Role of PAR-4 in ovarian cancer
Source: Oncotarget. 2015 May 27;6(26):22641–52. doi: 10.18632/oncotarget.4010 (PMC4673188; doi:10.18632/oncotarget.4010)
Supplement: Supplementary file 1 [file oncotarget-06-22641-s001.pdf]

SUPPLEMENTARY DATA

RESULTS

Effect of PAR-4 levels on cell apoptosis

The effect of PAR-4 on apoptosis of A2780 cells is assessed under basal condition or taxol treatment. Under basal condition, the downregulation or upregulation of PAR-4 doesn't influence the activity of caspase 3/7 compared to respective control by luminescent assay (Supplementary Figures S1A) and also the level of cleaved PARP by Western Blot (Supplementary Figures S1B). However, under taxol treatment, PAR-4 overexpression leads to an increase in cell apoptosis and in the opposite, PAR-4 silencing leads to decrease in cell apoptosis compared to respective control (Supplementary Figures S1A and S1B).

Effect of PAR-4 expression on GRP78 expression and secreted GRP78 in ovarian cancer cells

We have transfected SKOV-3 cells with control or PAR-4 plasmid and with control or PAR-4 siRNA to evaluate the effect of PAR-4 expression on GRP78 expression and secreted GRP78. In SKOV-3 cells, neither overexpression nor decreased expression of PAR-4 influence the total GRP78 protein expression (Figures A) or secretion of GRP78 is observed (Figures B) by Western blot.

Correlation between PAR-4 mRNA expression and relative membrane GRP78 expression in purified ovarian cells

We evaluate the expression of PAR-4 mRNA and membrane GRP78 in various ovarian cells purified from borderline tumour (n = 3), healthy (n = 13) and high grade serous cancer ovaries (n = 12). We find a significant correlation between PAR-4 mRNA expression and the proportion of GRP78 protein found on the cell surface ($n = 33$, $r = 0.475$ and $p < 0.01$).

SUPPLEMENTARY FIGURES

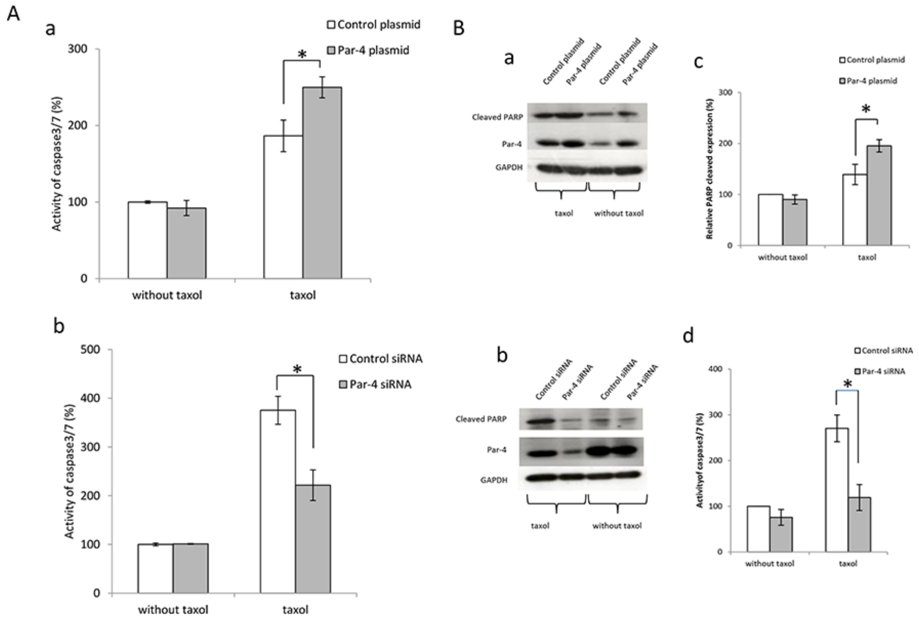

Supplementary Figure S1: Effect of PAR-4 level on cell apoptosis. A. Analysis of caspase 3/7 activity by luminescent assay. A2780 cells are transfected with control or PAR-4 expressing plasmid (a) and with control or PAR-4 siRNA (b) and treated or not with 100 nM of taxol. After 24 hours, caspase3/7 activity of cells is measured following the manufacturer instructions. **B.** Analysis of cleaved PARP by Western Blot. A2780 cells are transfected with control or PAR-4 expressing plasmid (a) and with control or PAR-4 siRNA (b) and treated or not with 100 nM of taxol. After 24 hours, cell extract is collected. Western Blot is performed and probed with anti-cleaved-PARP, anti-PAR-4 and anti-GAPDH antibodies. Bands of western blot are revealed by an ECL method, scanned and quantified by the Kodak 1D image analysis software. The cleaved-PARP band intensity is quantified and normalized to Actin and is expressed in percentage in function of control plasmid without taxol.

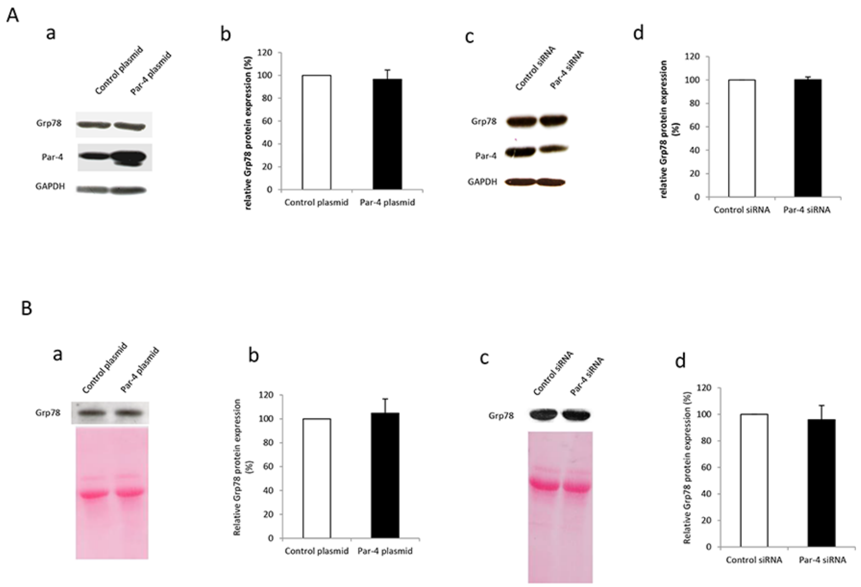

Supplementary Figure S2: Effect of PAR-4 expression on GRP78 expression and secreted GRP78 in ovarian cancer cells. A. Effect of PAR-4 on GRP78 expression. SKOV-3 cells are transfected with control or PAR-4 expressing plasmid (a, b) and with control or PAR-4 siRNA (c, d). 48 hours after transfection, cells are trypsizined and the expression of total GRP78 protein is analysed by Western Blotting analysis. Western blot is performed and membrane is probed with anti-GRP78 (Gl19), anti-PAR-4 and anti-GAPDH antibodies (a, c). Bands of western blot visualized by an ECL method are scanned and quantified by the Kodak 1D image analysis software. The GRP78 band intensity is normalized to Actin and is expressed in percentage in function of control plasmid. **B.** Effect of PAR-4 expression on secreted GRP78. SKOV-3 cells are transfected with control or PAR-4 expressing plasmid (a, b) and with control or PAR-4 siRNA (c, d). After 24 hours, the medium is changed and 1 ml of RPMI 1640 without FBS is added. 24 hours after, the medium is collected and concentrated with Centrifugal Fliter Units (UFC500324, Merck Millipore, Darmstadt, Germany). Western blot of secreted protein is performed and membrane is probed with anti-GRP78 (Gl19) antibody (a, c). Band intensity is quantified by the Kodak 1D image analysis software. Results are normalized with Ponceau (Sigma Aldrich, St Louis, MO, USA) staining membrane (b, d).

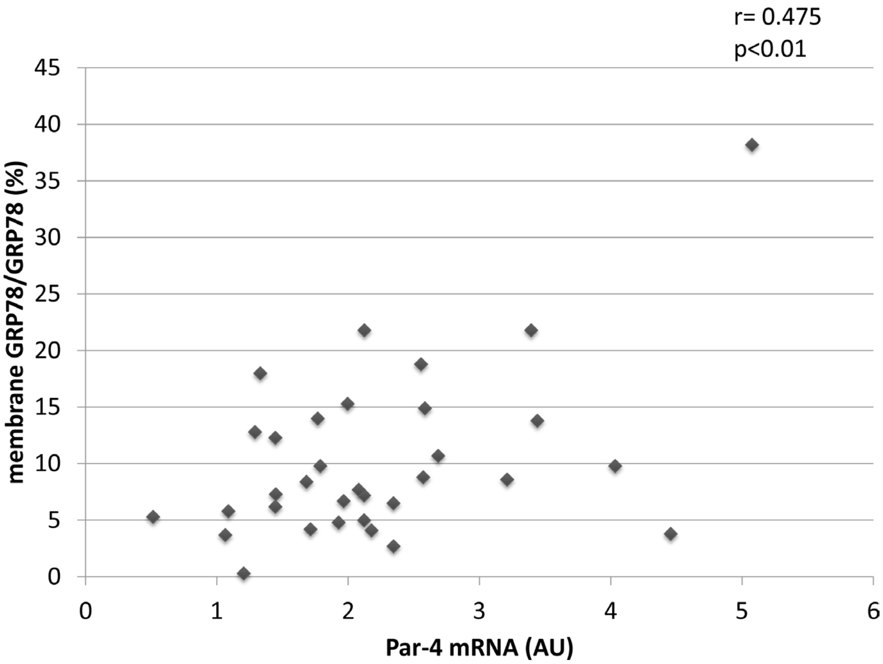

Supplementary Figure S3: Correlation between PAR-4 mRNA expression and membrane GRP78 expression in purified ovarian cells from high grade serous cancer, borderline tumour and healthy tissues or ascites. After purification of cells, a qPCR for PAR-4 and a cell ELISA for membranous GRP78 are performed. Two housekeeping genes are used: GAPDH and Cyclophilin A. A curve is established with PAR-4 qPCR results and membrane GRP78 expression results. Then, Pearson's correlation coefficient is used to show the correlation between PAR-4 and membrane GRP78 expression.
